# Virtual consultations for provision of care to people with chronic diseases/Consulta virtual para la atención a las enfermedades crónicas

**Published:** 2012-05-29

**Authors:** M.E. Pérez Cánovas, I. Llorente Gómez de Segura, M.D. Marrero Díaz, M.I. Fuentes Galindo, M. Cueto Serrano, A. Viñas Pérez

**Affiliations:** Tenerife Primary Care Administration, Santa Cruz de Tenerife, Spain; Nuestra Señora de la Candelaria University Hospital, Santa Cruz de Tenerife, Spain; Tenerife Primary Care Administration, Santa Cruz de Tenerife, Spain; Tenerife Primary Care Administration, Santa Cruz de Tenerife, Spain; Nuestra Señora de la Candelaria University Hospital, Santa Cruz de Tenerife, Spain; Nuestra Señora de la Candelaria University Hospital, Santa Cruz de Tenerife, Spain

**Keywords:** continuity of care, healthcare organisation, interprofessional relationships, continuidad de la atención al paciente, administración de los servicios de salud, relaciones interprofesionales

## Introduction

For the administration of healthcare, the autonomous region of the Canary Islands is divided into seven health regions, geographically corresponding to each of the islands. The health region of Tenerife is composed of a primary care unit (primary care administration) and two specialised care units, one based in each of the university hospitals on the island, the University Hospital of the Canary Islands (HUC) and Nuestra Señora de Candelaria University Hospital (HUNSC). Provision of specialist care is divided between two geographical zones, each of which is a catchment area for one of the hospitals and also has specialised outpatient centres.

A system for primary care (PC) electronic medical records (EMRs), called Drago AP, has been developed for the Canary Islands, and had been adopted across the entire region by 2010. On the other hand, an EMR system has not been yet introduced in specialised outpatient care.

The referral Endocrinology Unit for the centre and south of the Island (catchment of the HUNSC) carried out a detailed study of its provision of care through primary care and the characteristics of the consultations carried out. On the basis of the results, a reorganisation of healthcare delivery and internal measures for improvement were proposed. In relation to this process, the unit contacted primary care colleagues to identify mechanisms for collaboration.

## Description of the project

In 2008, in collaboration with the Endocrinology Unit of the HUNSC, given its particular interest in improving communication with PC, the use of the Drago AP system was extended to cover specialist outpatient appointments.

**Figure 1.**
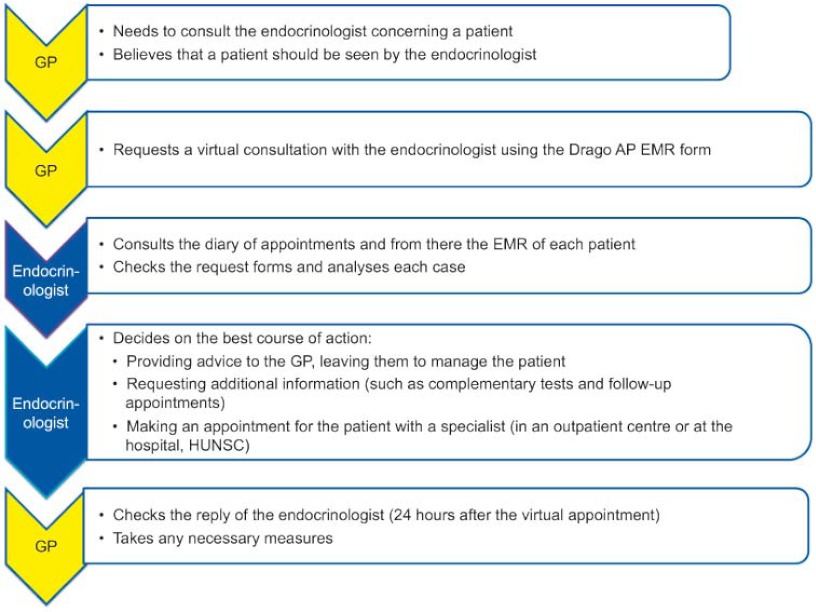
Care pathway in relation to virtual endocrinology appointments.

An appointment diary called “Virtual Endocrinology” was created within the specialities that the general practitioners (GPs) can access, and when a doctor requests a consultation with this speciality, a virtual appointment is made for the patient in this diary (Figure 1). The type of consultation that can be requested is not subject to pre-established criteria.

The endocrinologist, through the Drago AP EMR in their workplace, accesses the virtual diary, assesses the GP´s request, and responds through the same software using the existing referral form. There are three possible courses of action: they can address the GP´s request by providing advice on patient treatment or management, request additional information to complete their assessment, or make an appointment with a specialist in an outpatient centre or hospital departmentfor the patient’s condition to be assessed (the endocrinology unit contacts the patient to inform them about the appointment).

The Endocrinology Unit has undertaken to respond within 24 hours of the GP requesting a consultation. In practice, the agenda is available for appointments every day (from Monday to Friday).

The project was initially set up as a pilot in three health regions; the virtual endocrinology diary was developed and endocrinologists were trained in the use of the DRAGO-AP system, while the usual pathway for referrals was also maintained. The endocrinologists participating in the project made an effort to standardise their responses to GPs. Management from both levels of care as well as the endocrinologists were involved in presenting the project in the health centres.

## Results

The pilot lasted for 1 year (November 2008–December 2009). After this time, we observed that the number of consultations resolved through this system increased significantly, a progressive change in GPs’ reasons for requesting consultations, and also an increase in the satisfaction of the professionals with the tool in both levels of care. From February 2011, the project has been extended to all the health regions in the catchment area of HUNSC. Currently, all GP consultations with the Endocrinology Unit are carried out virtually. Moreover, the initiative is being extended to other specialities and health regions in the catchment area of the other hospital (HUC).

The adult catchment population (15 years of age or older) included in the project has now reached about 375,000 people (21 health regions with 242 staff doctors).

**Figure 2.**
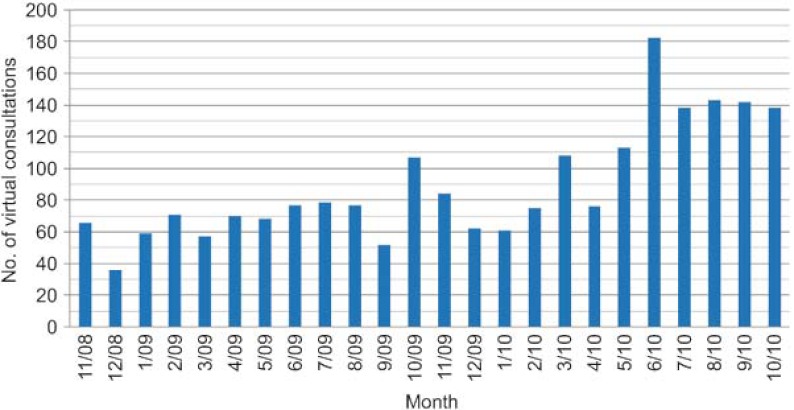
Trend in the number of virtual consultations per month over the first 2 years (absolute number).

**Figure 3.**
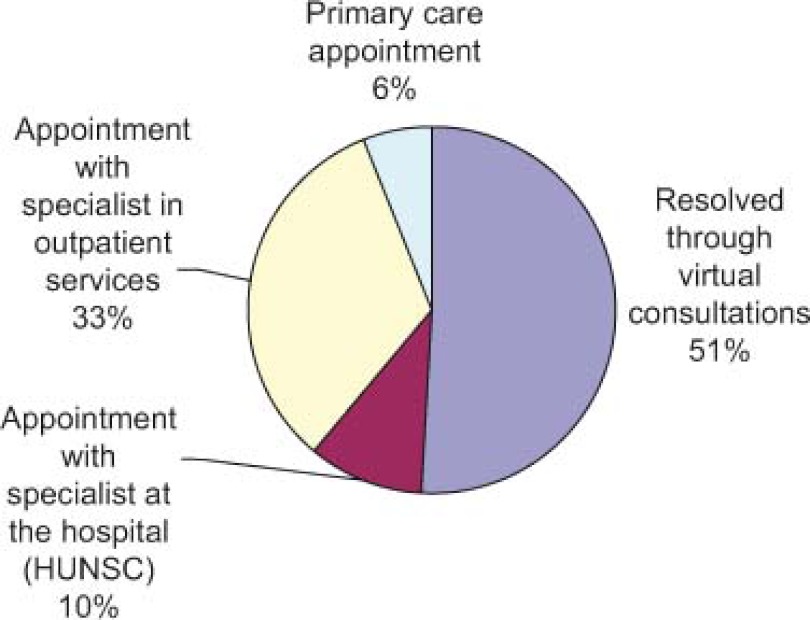
Results of the virtual consultation (%).

**Figure 4.**
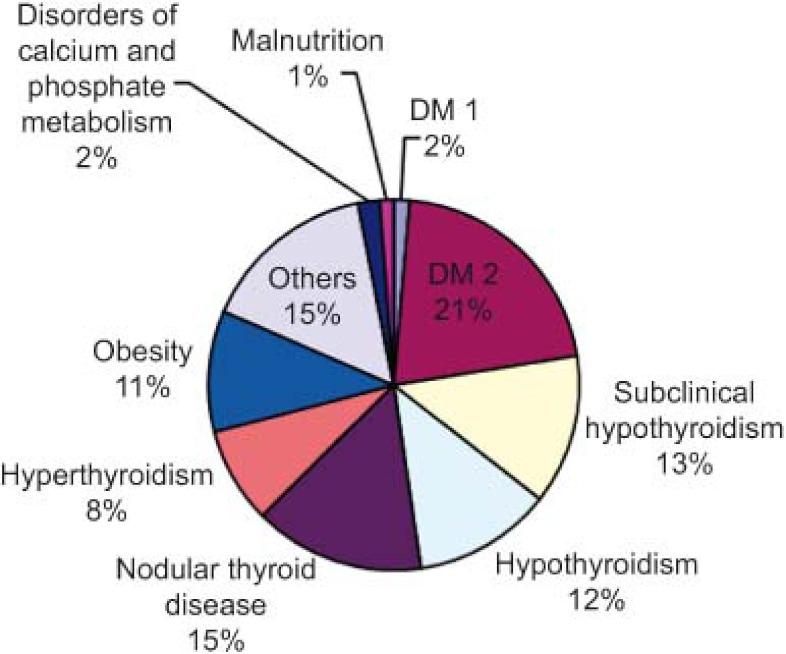
Reasons for requesting a virtual consultation with the endocrinologist, expressed as a percentage of the total.

A total of 2141 virtual consultations were made over the first two years (Figure 2). Of these, queries were resolved through the virtual consultations in 51% of cases and 43% of patients were given appointments with specialists, all within a reasonable time (Figure 3). Requests for consultations were most commonly made in relation to the following diseases, in decreasing order of importance: diabetes (DM), thyroid diseases and obesity (Figure 4).

## Discussion

Some problems remain to be solved including issues related to insufficient development of the IT system. Specifically, it is still necessary for the specialists to use the primary care medical records, and some of the related tasks are not automated (for example, checking whether a request has been answered). Further, given that the desire to extend the system to other specialities, it is going to be necessary to have software that permits the integration of all the available complementary tests in the EMR to facilitate the consultation process. We also have detected a need for specialists involved in the project to have skills for managing their relationship with GPs.

Despite these factors, the project has created a channel of communication between primary and specialised care (to which patients are referred) that is efficient and effective and has enabled patients to be provided care in the most suitable place depending on their condition (primary care, specialist outpatient centres or the hospital), avoiding unnecessary consultations and travel for patients. In addition, we have observed an improvement in the ability of GPs to solve endocrinological problems independently, due to the learning that takes place with this system.

The Endocrinology Unit has reorganised its provision of care, increasing the services provided to its catchment area, without imposing referral criteria and with a clear willingness to collaborate with primary care. Among other benefits, this has led to a significant decrease in the waiting time to see a specialist.

## Conclusions

It is possible to improve coordination between levels of care with the necessary motivation, even with imperfect tools, as long as there are shared goals focused on patient needs and the role of primary care.

## Conference abstract Spanish

## Introducción

Desde el punto de vista sanitario, la Comunidad Autónoma de Canarias está formada por siete áreas de salud, que se corresponden geográficamente con cada una de las islas. El área de salud de Tenerife se compone de una Gerencia de Atención Primaria (GAP) y dos Gerencias de Atención Especializada en cada uno de los dos Hospitales Universitarios que existen (Hospital Universitario de Canarias (HUC) y Hospital Universitario Nuestra Señora de Candelaria (HUNSC)). La asistencia sanitaria especializada está sectorizada en función del área geográfica en dos zonas, dependientes de cada hospital, teniendo, cada una, en el ámbito extrahospitalario, los Centros de Atención Especializada (CAE).

En la Comunidad Canaria se ha desarrollado un sistema propio de Historia Clínica Electrónica de Atención Primaria (HCE-AP), denominada DRAGO-AP e implantada en el global de nuestra comunidad desde el año 2010. No se ha implantado un sistema de historia electrónica en atención especializada extrahospitalaria.

El Servicio de Endocrinología de referencia para la zona centro y sur de la isla (HUNSC) había realizado un estudio detallado de la situación de su oferta asistencial hacia AP y de las características de las consultas atendidas. A partir de ello se planteó una reorganización asistencial del modelo de consulta y propuestas de mejora internas. En relación con este proceso entraron en contacto con Atención primaria para buscar mecanismos de colaboración.

## Descripción de la experiencia

En el año 2008, junto al Servicio de Endocrinología del HUNSC y por su especial interés en mejorar la comunicación con AP, extendimos el uso de Drago AP al ámbito de los CAEs.

**Figura 5.**
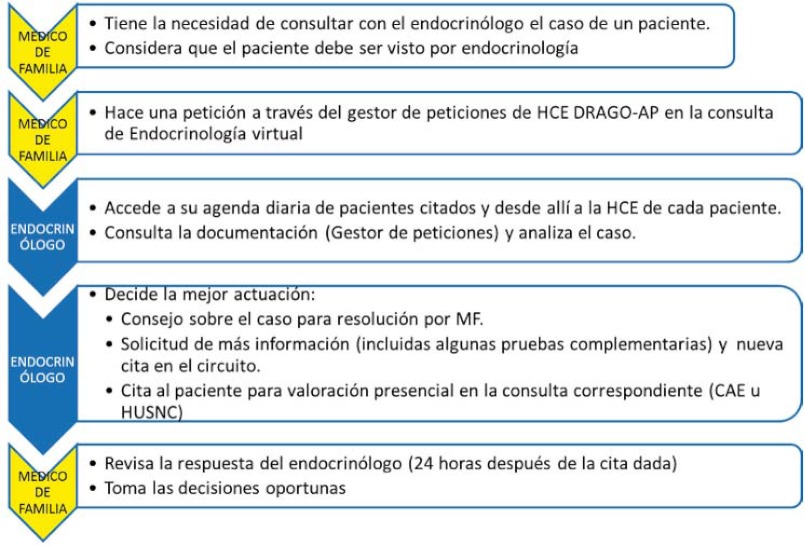
Circuito asistencial en la consulta virtual de Endocrinología.

Se definió una agenda de consulta denominada “Endocrinología virtual”, dentro de la cartera de especialidades que tienen los Médicos de Atención Primaria (MAP) a disposición, de manera que cuando el médico solicita una consulta a este servicio, se genera una cita al paciente en esta agenda (Figura 5). El tipo de consulta que se puede realizar no está sujeto a criterios preestablecidos.

El endocrinólogo, a través de un acceso a HCE Drago-AP en su lugar de trabajo, accede a su agenda virtual y valora la consulta del MAP, respondiendo siempre a través de la misma aplicación en el documento de interconsulta existente.

Las respuestas pueden ser de tres tipos: resolución de la consulta mediante consejo terapéutico o de manejo del paciente, solicitud de información adicional para completar la valoración, o cita en el CAE o en consulta específica hospitalaria para realizar una valoración presencial del paciente (el servicio de endocrinología contacta con el paciente para informarle de la cita).

El compromiso del Servicio de Endocrinología incluye la respuesta al MAP en 24 horas a partir de la cita. La disponibilidad actual de agenda es diaria.

El proyecto se puso en marcha inicialmente en tres zonas básicas de salud, se formó a los endocrinólogos en el uso de Drago-AP, se diseñó la agenda de endocrinología virtual y se mantuvo la vía ordinaria de interconsulta. Los endocrinólogos que participan en el proyecto hicieron un esfuerzo para homogeneizar las respuestas que se darían a AP. Para la presentación del proyecto en los centros se implicaron ambas gerencias así como los endocrinólogos.

## Resultados

El pilotaje duró un año (noviembre 2008-diciembre 2009). Después de ese tiempo se encontró una evolución positiva significativa en el número de consultas resueltas por este sistema, un efecto de cambio progresivo en los motivos de consulta, así como satisfacción de los profesionales de ambos ámbitos con la herramienta. Desde el mes de febrero de 2011 el proyecto funciona en todas las Zonas Básicas de Salud (ZBS) que tienen de referencia al HUNSC. Y en la actualidad el 100% de las consultas al Servicio de Endocrinología se realizan por vía virtual. Por otro lado, la experiencia está en fase de extensión a otras especialidades y a las zonas de salud que tienen como hospital de referencia el Hospital Universitario de Canarias.

La población adulta (de 15 años en adelante) de referencia incluida en el proyecto está en torno a las 375.000 personas (21 ZBS con 242 médicos de plantilla).

**Figura 6.**
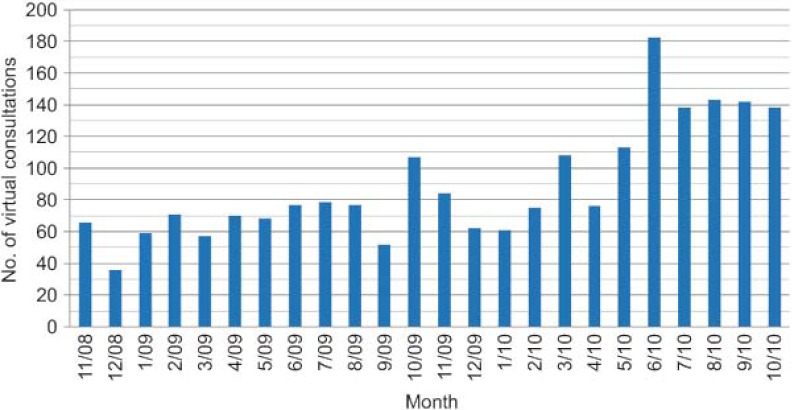
Evolución del número mensual de consultas a lo largo de los dos años (número absoluto).

**Figura 7.**
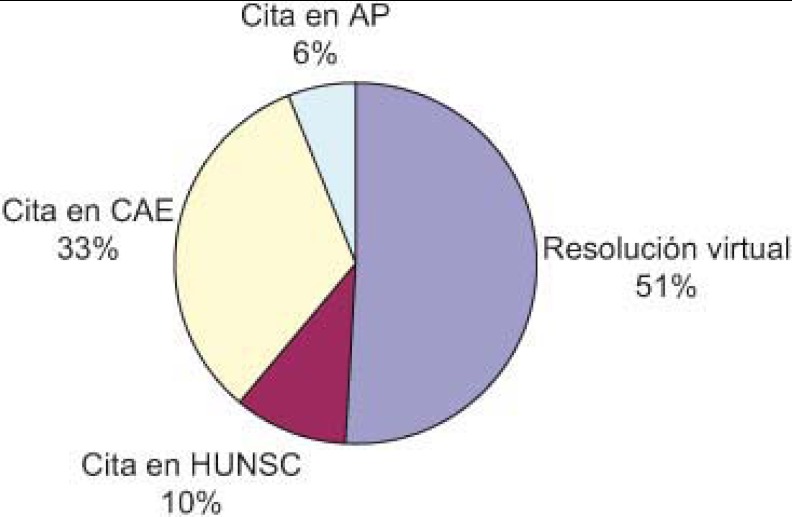
Resultado de la consulta virtual (%).

**Figura 8.**
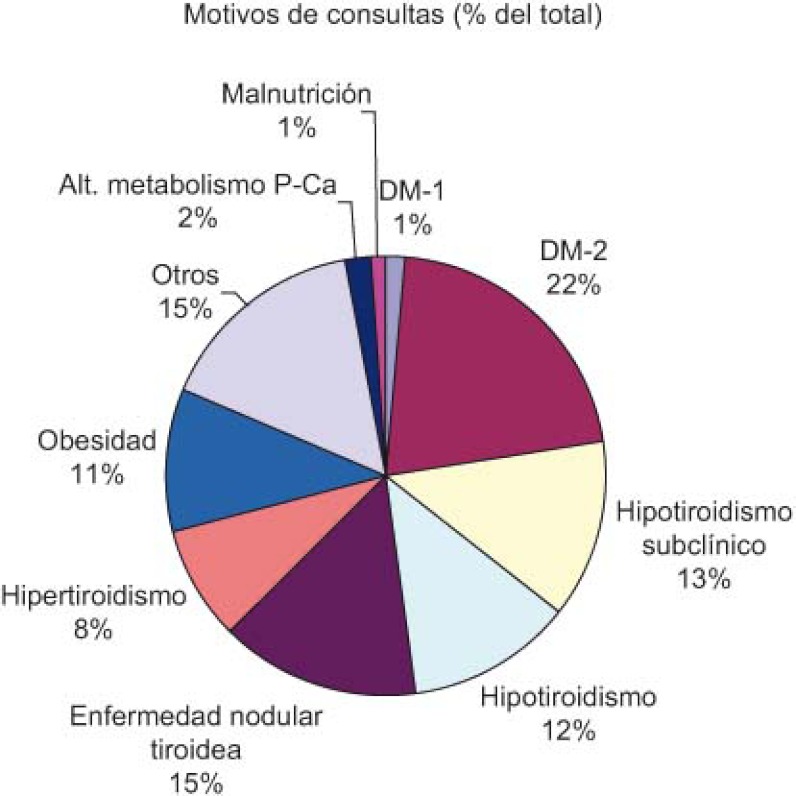
Motivos de consulta al endocrino virtual expresado en porcentaje del total.

A lo largo de dos años, el total de consultas virtuales que se realizaron fueron de 2.141 (Figura 6), de ellas se resolvieron virtualmente el 51% y el 43% se citaron por parte de AE en un tiempo razonable (Figura 7). Las patologías más consultadas han sido, por orden: diabetes, enfermedades del tiroides y obesidad (Figura 8).

## Discusión

Como problemas aún pendientes de resolver se encuentran los relacionados con un desarrollo insuficiente de las herramientas informáticas. Esto supone que todavía es necesario que los médicos de AE usen la historia de AP, y algunas tareas relacionadas con el proceso no están automatizadas (por ejemplo, la comprobación de que hay respuesta). Además, al tener el objetivo de su ampliación a otras especialidades, se hace necesario disponer de software que permita la integración de todas las pruebas complementarias en la HCE para facilitar el proceso de consultoría. Por otro lado hemos detectado la necesidad de que los profesionales de AE destinados al proyecto tengan habilidades para gestionar la relación con los MAP.

Este proyecto ha permitido crear un canal de comunicación entre atención primaria y el servicio de atención especializada de referencia eficaz y eficiente, y que la atención al paciente se realice en función de su patología, en el lugar más adecuado (AP, CAE, HUNSC), evitando consultas y desplazamientos innecesarios a los pacientes. Además se ha observado una mejora de la capacidad de los MAP para resolver independientemente los problemas endocrinológicos, derivado del aprendizaje que supone el sistema.

El Servicio de Endocrinología ha realizado un proceso de reorganización de su oferta asistencial incrementando los servicios al área, sin imposición de criterios de derivación y con una clara vocación de colaboración con atención primaria. Esto ha redundado en una disminución significativa de la demora para obtener consulta.

## Conclusiones

Con la motivación adecuada es posible mejorar la coordinación entre ámbitos asistenciales, incluso con herramientas imperfectas, teniendo claro un objetivo común centrado en las necesidades de los pacientes y en el papel de la atención primaria.

